# Adolescents, parents and teachers’ perceptions of risk and protective factors of substance use in Moroccan adolescents: a qualitative study

**DOI:** 10.1186/s13011-018-0169-y

**Published:** 2018-09-10

**Authors:** Hicham El Kazdouh, Abdelghaffar El-Ammari, Siham Bouftini, Samira El Fakir, Youness El Achhab

**Affiliations:** 1 Laboratory of Epidemiology, Clinical Research and Community Health, Faculty of Medicine and Pharmacy of Fez, University of Sidi Mohamed Ben Abdellah, Fez, Morocco; 2 Regional Centre for Careers Education and Training of Fez-Meknes, Fez, Morocco

**Keywords:** Adolescents, Morocco, Qualitative study, School, Socio-ecological model, Substance use

## Abstract

**Background:**

Substance use in adolescents is a global public health concern that continues to draw attention from academics, policy experts, and government officials. In Morocco, few studies have investigated the influencing factors of substance use in adolescents. Here, we aimed to fill this gap and to better understand factors that protect or influence substance use in adolescents.

**Methods:**

We conducted a qualitative study using focus group discussions. The semi-structured interview guides were based on the socio-ecological model as a theoretical framework to explore perceptions of students, parents, and teachers regarding substance use risks and protective factors in adolescents. Data from each group were audio-recorded, transcribed, and analyzed using thematic analysis.

**Results:**

From May to July 2016, 17 focus group discussions were conducted at two middle schools in Taza city, Morocco, which included 8 groups of 7 adolescents (28 boys and 28 girls) aged 14 to 16 years, 5 groups of parents (5 females and 21 males), and 4 groups of teachers (13 males and 5 females). Thematic analysis resulted in six common themes that represented the most salient perceived risk and protective factors regarding substance use among adolescents: perceived benefits of substance use, awareness and beliefs, family influence, peer influence, easy accessibility of substances, and social norms.

**Conclusions:**

Our results demonstrate that multilevel prevention programs in adolescents should address influencing factors from the individual to the societal level, including social norms and the government’s policy toward substance use. Health education programs included as part of the school curriculum can contribute to promoting awareness and reducing risky behaviors of Moroccan adolescents.

## Background

Adolescence is a time of physical, psychological, and social and emotional changes. At this stage of life, adolescents adopt behaviors that can lead to various health risks, including substance use [1]. For adolescents, substance use tends to be acquired through experimentation and curiosity, particularly through peers [2].

Alcohol, tobacco, and illicit drugs are the most used substances by adolescents worldwide, and these substances share similar patterns of use [3, 4]. Substance use is a leading cause of preventable morbidity and mortality among youth; indeed, more cases of death, disease, and disability are caused by substance use than by any other preventable health condition [5]. In addition, substance use is associated with many psychological, social, and economic complications, including depression, suicidal behavior, crime, and the global financial burden of drug abuse [6, 7].

Early involvement of substances increases the possibility that addiction will develop [8]. Substance use in adolescents is common in both low- and high-income countries [9, 10], and Morocco has shown both increased prevalence and early initiation of substance use in adolescents [11, 12].

Risk factors that potentially influence adolescents to use substances include experimentation, lack of awareness, poor parental monitoring, peer and family influences, gang affiliation, psychological problems, absenteeism, and below-average grades [13, 14]. Having a family member who uses substances can also be a risk factor; however, enjoyment and curiosity are cited as major influences in decisions by adolescents to use substances [15].

Protective factors against substance use have also been identified. These include prosocial peers, home support, school support, self-awareness, peer caring relationships, and community support [16]. In school settings, school attendance, parental or guardian connectedness, peer support at school, and parental supervision have been shown to be protective factors for adolescent health [17]. Religiosity can also keep teenagers from taking substances, even when they are experiencing difficult situations [18, 19].

An effective substance prevention program in adolescents requires clear identification of contextual risks and protective factors. Thus, the socio-ecological model has long been recommended to guide public health intervention research [20]. The principle of this theoretical framework is that the contextual environment (e.g., home, school, community) interacts with the individual to promote healthy behaviors or to create unhealthy ones, such as the use of substances [20]. This model allows exploration of complex interactions between multiple factors that influence risky behaviors in adolescents, thus presenting ways to promote healthy behaviors [21]. In the socio-ecological model [21], factors influencing the use of substances among adolescents have been categorized into four levels: *individual*, *relationship*, *community,* and *societal*.

In Morocco, the Mediterranean School Survey Project on Alcohol and Other Drugs, conducted in 2009 and 2013 among 15- to 17-year-olds, revealed that substances were used and started at an early age [12]. Similarly, results from the 2010 and 2016 Global School Health Surveys demonstrated an increasing prevalence of substance use among adolescents aged 13 to 15 years, especially regarding use of tobacco and cannabis [11, 22]. Another study conducted in the North Central Region of Morocco among 11- to 23-year-old school students reported a 16.1% lifetime prevalence of smoking and a 9.3% prevalence of using psychoactive substances. Specifically, cannabis was the most used substance (8.1%) followed by alcohol (4.3%) [23]. These studies show that tobacco and cannabis are the most used substances in adolescents compared to alcohol and other illicit drugs. This can be explained by the prohibition and disapproval of alcohol and illicit drug use by the religious, social and sometimes legal systems of our country. On the other hand, Morocco, it is one of the largest producers of cannabis resin in the world [24], making cannabis easy and inexpensive to obtain by teenagers.

Studies of factors that influence substance use in Moroccan adolescents are scarce, with most focusing on only individual and/or family factors [12, 13, 23]. This study seeks to fill this gap and this lack of data on contextual risk and protective factors of substance use (especially regarding use of tobacco and cannabis) in Moroccan adolescent. Here, we used a socio-ecological approach and focus group discussions (FGDs) with multiple-category design to obtain accurate descriptions of perceived risks and protective factors of substance use in Moroccan adolescents. Having a multiple-category design allows views of different groups to be explored in a similar category or in different categories [25]. For this study, we examined views of three different groups (adolescents, parents, and teachers) [26]. These results may aid in the development of more effective interventions.

## Methods

### Study participants and procedures

To explore perceived contextual risks and protective factors of substance use, we conducted 17 FGDs with 100 participants in Taza city, Morocco, from May to July 2016. We had 8 groups of adolescents (28 males and 28 females, 14–16 years old) who were recruited from two middle schools (disadvantaged and advantaged according to socio-economic level). From each school, adolescents from the last year of middle school were selected based on purposive sampling to obtain groups balanced across age, sex, and substance use behaviors. This age was selected because their educational programs include studies related to health risk behaviors. In addition, we had 5 groups of parents of adolescents (5 females and 21 males) who were included based on their voluntary participation. Teachers of disciplines concerned with health risk behaviors were randomly selected from included schools and grouped into 4 FGDs (13 male and 5 female teachers). Participant characteristics are shown in Table 1. We arranged participants into single-sex focus groups to respect the sociocultural norms of Morocco. Sampling continued until data saturation was reached.

**Table 1:** Participant characteristics

	Student Group (*n* = 56)		Parent Group (*n* = 26)		Teacher Group (*n* = 18)	
	Male	Female	Male	Female	Male	Female
Age range, y	14–16	14–16	40–60	40–60	30–60	30–60
No. of focus groups	4	4	4	1	3	1
No. of participants per focus group	7	7	5 or 6	5	4 or 5	5
No. of participants (%)	28 (50)	28 (50)	21 (80.8)	5 (19.2)	13 (72.2)	5 (27.8)

Privacy and confidentiality were maintained during interviews, which were conducted by two researchers. A moderator gave appropriate information about the study aims before the interview, and participants were informed about the tape-recording procedure and confidentiality. A silent observer made notes on non-verbal behaviors of individuals and group interactions. Before the FGDs were closed, participants were invited to add to or clarify their opinions. Each FGD lasted approximately 45 to 60 min.

### Data collection instruments

Participants completed a brief, structured one-page questionnaire to record demographic characteristics. Qualitative data were gathered through FGDs using semi-structured interview guides. Three different sets of open-ended questions (adapted for adolescents, parents, and teachers) were used to explore the perceived risks and protective factors for substance use in adolescents at the individual, relationship, community, and societal levels (Table 2). Our interview guides were developed by the research team based on the socio-ecological model as a theoretical framework [21]. Interview questions were tested on small groups of each category of participants to identify wording issues.

**Table 2:** Sample of open-ended questions in focus group discussion guides

Preamble	
After the team members introduced themselves, the moderator gave appropriate information about the study aims, and participants were informed about the tape-recording procedure and confidentiality. Then, a clear definition was communicated to participants regarding substances: “The three commonly used psychoactive drugs: alcohol, cigarettes, and illicit drugs that produce changes in mood, thinking, feeling, and/or behavior and can cause dependence.”	
Open-ended questions used to map discussions and to identify substance use behaviors and the risk and protective factors in adolescents	
Adolescent questions: 1. Can each one of you tell us about his (her) information about the dangers of substance use and whether he (she) uses any type of substances or has used them before? 2. Can you tell us what are the (socio-ecological) factors that push teens to start using substances? 3. Can you tell us what are the (socio-ecological) factors that prevent teens to start using substances? Parent questions: 1. Can each one of you tell us about his or her knowledge about the dangers of substance use and whether your children or teenagers in general use them? 2. In your opinion, what are the (socio-ecological) factors that push your children or teens in general to start using substances? 3. In your opinion, what are the (socio-ecological) factors that prevent your children or teens in general to start using substances? Teacher questions: 1. Can you tell us about the level of substance use among students and teenagers in general today? 2. In your opinion, what are the (socio-ecological) factors that push teens to start using substances? 3. In your opinion, what are the (socio-ecological) factors that prevent teens to start using substances? 4. Can you tell us what the role of the school is in either increasing or decreasing substance use in adolescents?	

### Ethical approval and consent to participate

The study was approved by the Faculty of Medicine and Pharmacy of Casablanca Research Ethics Committee and the National Control Commission for the Protection of Personal Data (A-RS-193-2015). Written informed consent was obtained from all participants before study enrollment. For adolescent participants, written informed consent was obtained from their parents or legal guardians, with verbal consent from each adolescent participant. Participants were informed that they could withhold information. The names of participants were separated from the transcripts and field notes and kept in a private place where only the leading author had access. Only the principal investigators had access to the full tapes.

### Data analyses

All sessions were audiotaped, transcribed verbatim, and translated into English. All transcripts were checked for accuracy through listening to recordings when reading transcripts. Data sets based on adolescent, parent, and teacher discussions underwent thematic analysis [27], which allows repeating themes to be recognized across a data set. The inductive thematic analysis technique was used to explore accounts among participants without trying to fit these into a preexisting coding frame [27]. In addition, a semantic level of thematic analysis was used [27].

Data were analyzed using six phases of thematic analysis as recommended by Braun and Clarke [27] The coding process was iterative; to ensure transparency and reliability, and all transcripts were coded independently by the two researchers. Differences were then discussed and resolved to refine codes and identify key themes emerging from the data. The overreaching themes were then categorized according to the socio-ecological model [21], allowing us to explore several contextual factors influencing attitudes, beliefs, and practices associated with substance use in adolescents.

## Results

Participant statements mainly focused on factors influencing tobacco use (including cigarette smoking, water pipes, and smokeless tobacco) and cannabis use (sepsi pipe and edible and smoked cannabis products). Some participants also commented on use of alcohol and other drugs.

**Fig. 1:**
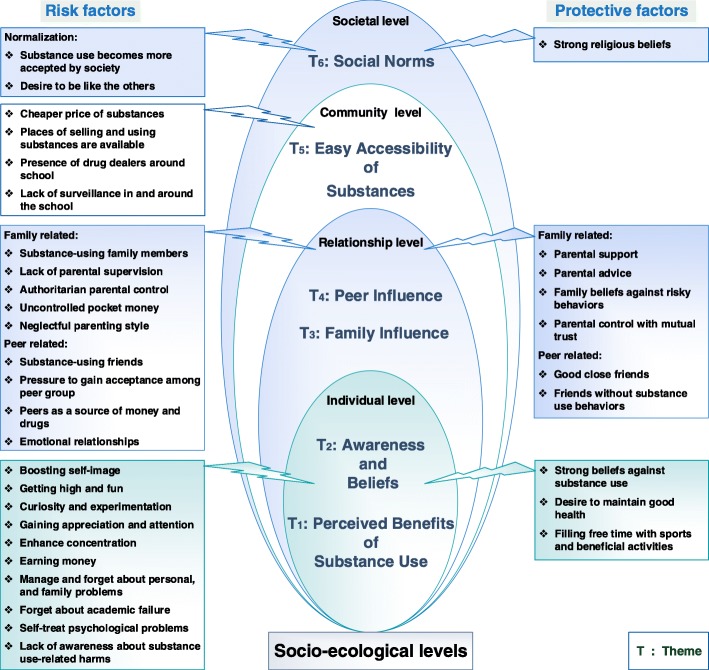
Diagram showing emerging themes and factors influencing substance use in Moroccan adolescents within the socio-ecological model

Our analyses identified six common themes regarding substance use among the four categorical levels **(**individual, relationship, community, and societal)**:** perceived benefits of substance use, awareness and beliefs, family influence, peer influence, easy accessibility of substances, and social norms. Table 3 shows illustrative quotes from different focus groups in support of each theme and sub-theme. In addition, Fig. 1 illustrates how the themes (factors influencing substance use in adolescents) fit within the socio-ecological model levels.

**Table 3:** Themes and examples of statements

Theme	Example	Illustrative Statements
Perceived Benefits of Substance Use	Ex. 1	***G1:*** *“Sometimes adolescents consider smoking as one of the ways of imposing themselves and showing off in front of the others; they smoke at this very early age, which is why we wonder what these youngsters would do once they grow old.”* ***F1:*** *“There are also those feelings of self-awareness from the part of those youngsters.”* ***F.T1:*** *“According to me, those youngsters find that these behaviors and attitudes are complementary to their character and a way of confirming their manhood.”*
	Ex. 2	***G2:*** *“The reason behind the consumption of drugs sometimes is to look popular and to feel loved by their friends; sometimes the girl may start smoking only to get famous and well-known among her peers.”* ***F2:*** *“…a kind of fashion and fame!?!”* ***F.T2:*** *“Drug consumption and smoking can be sometimes an act of impressing others because it is highly regarded that smoking is part of the local tradition of displaying virility.”*
	Ex. 3	***B1:*** *“When you try it for the first time, it surely takes one to a different world; the use of drugs gradually increases day after day.”* ***F3:*** *“Some adolescents strongly believe that the use of drugs makes one active, and it is the only source of the feeling of high ecstasy.”* ***M.T1:*** *“When adolescents use drugs, they find himself in a state of indescribable pleasure, the feeling of euphoria; thus, the adolescent keeps on consuming drugs on a daily basis until they suddenly find themselves addicted, which makes it hard for them to let it go.”*
	Ex. 4	***G3:*** *“The period of adolescence plays a major role in creating all these problems and instills acts of carelessness within teenagers.”* ***M1:*** *“There are also the effects of adolescence and natural motivations.”* ***M.T2:*** *“In this period, students are exposed to smoking and even drug abuse; I think … the reason is the lack of awareness, and a teenager does not have enough sense of responsibility.”*
	Ex. 5	***B2:*** *Interviewer: (what was the factor that led you to smoke at first?)* *“I just wanted to try it with friends … out of curiosity.”* ***F4:*** *“This first experience with friends is extremely dangerous, the desire to experiment and explore at the beginning, and after that it becomes a habit.”* ***M.T3:*** *“He just wants to taste these substances (try these taboos), and when he tries, he’s going to be at risk for addiction ... (although he just tried).”*
	Ex. 6	***B3:*** *“Some adolescents have psychological problems; they believe that the only solution to cope with their problems is to use different substances.”* ***F5:*** *“What drives teens to use drugs are psychological problems like shyness, lack of self-confidence…”* ***F.T2:*** *“Some psychological problems are behind adolescents’ substance use, like having a weak personality… or living in an uncomfortable atmosphere.”*
	Ex. 7	***G2:*** *“Some students suffer from poverty and compare themselves with other people who live in good homes and excellent conditions; they want to try drugs to forget their living reality.”* ***G4:*** *“I know a poor girl who used to be my classmate whose parents decided to get divorced, so she was living with her grandmother ... all these problems prompted her to use drugs.”* ***F6:*** *“Social factors such as poverty, unemployment and vulnerability, the housing crisis, such as shantytowns, and the labor crisis … lead some people to use substances in order to forget their problems.”* ***F6:*** *“There are also many causes like domestic problems, including divorce of parents.”* ***F.T1:*** *“Adolescents tend to escape and avoid their personal, economic, and social problems by using drugs.”* ***F.T3:*** *“I believe that there are many family problems such as divorce that place adolescents in this situation.”*
	Ex. 8	***G3:*** *“The problem of dropping out of school can cause adolescents’ substance use.”* ***M2:*** *“Study failure and dropping out of school may also be a cause of drug use in adolescents.”* ***F.T2:*** *“School failure can lead students to drug use.”*
Awareness and Beliefs	Ex. 9	***B4:*** *“For example, my father used to smoke and my brother does so ... but I don’t smoke because it is very harmful.”* ***G5:*** *“The media and schools also play an important role in health awareness through some awareness programs about the dangers of smoking and drugs. Unfortunately, this type of awareness is scarce in our context.”* ***F7:*** *“The morals that I instill in my children keep them away from using drugs.”* ***M3:*** *“Awareness through media, the Internet, and school sensitization campaigns against substances use for example.”* ***F.T2:*** *“The school is working to reduce these risky behaviors such as smoking, drug taking. However, it is ineffective, given the widespread of the phenomenon or sometimes the lack of monitoring, especially outside of school.”* ***M.T4:*** *“The curriculum must graduate a good citizen, and therefore these behaviors should be given great importance in terms of time and attention by the officials; nevertheless, such things are missing in our system.”*
	Ex. 10	***G3:*** *“Some adolescents are from families that are illiterate and unaware about the drug-related harms, in addition to the negative impact of the media.”* ***G6:*** *“Even in the media, adolescents notice that the actors smoke and use drugs, so they tend to imitate them.”* ***F8:*** *“The school no longer has a good impact because many other interests like the Internet have emerged, and the student has become uninterested in school, which led to a lack of awareness about drug-related harms.”* ***F3:*** *“Some teenagers are influenced negatively by some bad models that exist in the media like an athlete, star, actor ... and they try to imitate them.”* ***F.T2:*** *“They don’t care of the several harms caused by smoking and drug use.”* ***M.T4:*** *“One reason for these behaviors is the absence of awareness among parents. Also, our curricula don’t give much importance to these risky behaviors in terms of time and consideration by the officials, which contributes to a degradation of the awareness.”* ***M.T5:*** *“It is in fact the huge impact of the media outlets that misguides the adolescents by making them think that it is a form of social civilization!?!”*
	Ex. 11	***B5: “*** *I think that drugs are not useful. For example, I like to see myself well-built and without diseases. If I use cigarettes or drugs, they will harm my health. So, I don’t use them and I will not use them.”* ***B6:*** *“I have some friends who smoke but I don’t smoke. (Why?) I don’t want to because when you grow old you will lose all your money in addiction; I believe that cigarettes have no significance in solving or forgetting problems, and they only harm health and waste your money.”* ***B7:*** *“Sometimes when an adolescent has a father who consumes drugs, he sees that he suffers from diseases, so he doesn’t want to be like him and undergo the same consequences.”* ***F3:*** *“Some teenagers take lessons through one of the parent or the family member who used these substances and is in poor health because of these drugs.”* ***M.T2:*** *“First, they may have convictions because of an education they have had from their parents. This is why you can find a teenager whose father is a smoker, but he does not smoke and always criticizes his father. Because he gave him a negative image and he shows himself in bad situation.”*
	Ex. 12	***G3:*** *“There are those who fill their free time with useful things to avoid bad behaviors. For example; sports, because someone who practices sports must follow a special diet and get away from drugs.”* ***M4*** *: “Some teens have a healthy adolescence and have a high interest in studying, which keeps them far from drug use and addiction.”*
Family Influence	Ex. 13	***B3:*** *“If the father smoked and there was pressure on the teenager, he would say why I do not smoke like my father to forget about these problems.”* ***M4*** *: “Parents don’t care about their children; they don’t give them time to talk to them about their problems. Consequently, they begin to use drugs to forget. In addition, you can find some parents using cigarettes in front of their children.”* ***M.T5:*** *“In my opinion, the education at home and especially if it is free of religion, ethics, and communication between parents and children, will make adolescents vulnerable to being involved in such behavior.”*
	Ex. 14	***G8:*** *“We find that, for the girl who consumes drugs, her parents don’t care about her and don’t control her well. For example, she goes out at 8 in the morning, even if she doesn’t have a class or she isn’t going to go to class and stays out with addicted teenagers. So, she starts taking drugs.”* ***G9:*** *“Parents don’t control the time schedule of their children when they enter school and when they come out.”* ***F10:*** *“Absence of control by the family.”* ***F11:*** *“When a child feels ignored by parents, he is encouraged to take these substances.”* ***M.T2:*** *“I think bad parenting, when an adolescent sees that no person in his family gives him importance or controls him, he can do anything like smoking, consuming drugs,* etc.*”*
	Ex. 15	***B8:*** *“One student in our class drinks alcohol; his father gives him everything, but he wants to deviate because he has money. Excessive and unchecked pocket money leads to risky behaviors.”* ***B3:*** *“The other factor, I think, is the lack of parents’ control, especially when they give them money and don’t ask how they spend it.”* ***F12:*** *“The reason for me is the availability of money in an excessive and uncontrolled way.”* ***F4:*** *“Too much pocket money can be a cause of drug abuse, and its absence may lead to the same problems indirectly.”* ***F.T2:*** *“Some teenagers get ample money to cover their needs without parental control.”* ***M.T5:*** *“Giving children money without supervision or control.”*
	Ex. 16	***G10:*** *“As they say, my parents explained to me that drugs are bad and harmful.”* ***B2:*** *“My parents know that I don’t smoke and they trust me. The money with which I could buy drugs I use it to buy something else healthy.”* ***G11:*** *“The presence of control within the family as well as mutual trust between parents and children.”* ***M5*** *: “Parents should monitor their children and know who is accompanying them as friends.”* ***F3:*** *“Education and control of children since childhood within the family represent a protective factor.”* ***F.T4:*** *“Dialogue, understanding, and the attention given to an adolescent within the family, to know his problems and his needs, can keep adolescents away from this risk.”*
Peer Influence	Ex. 17	***G12:*** *“Bad company; sometimes the student who smokes begins to urge other teens to smoke and tell him you cannot smoke, you are not a man.”* ***B9:*** *“What made you try? We played at night with each other, but after that we left playing and started meeting in the day to smoke. We were 6 people, now two people are not smokers and the others are addicts.”* ***M5*** *: “I think the cause is bad friends and free time.”* ***F13:*** *“Adolescents learn from each other; the cause is the bad companions; they try to involve good boys and so on.”* ***M.T2:*** *“All this is linked to the so-called bad companions. Parents, for example, do not pay attention to their children if they go with some bad teenagers who use these substances.”*
	Ex. 18	***G4:*** *“One girl was in relation with one boy who used all kinds of drugs ... she saw how relaxed he was when he smokes; thus, she began using drugs and becomes addicted like him.”* ***F.T2:*** *“For female adolescents, emotional relationships and encounters with male adolescents have a significant impact on the use of cigarettes, drugs, and alcohol, especially in their talk about ecstasy.”*
	Ex. 19	***B10:*** *“I know how I choose my friends, and I had awareness before I reached this age because it is very dangerous.”* ***G13:*** *“I choose my companions; they should be good and I do not accompany the bad girls. At the beginning of the year, you can spend a week with your friend to know if she has bad behavior or not.”* ***M3:*** *“They choose friends who are smart and who understand things quickly, and those never used drugs.”* ***M.T6:*** *“They don’t accompany friends who use drugs and they don’t go to the places where the drugs are used.”*
Easy Accessibility of Substances	Ex. 20	***B11:*** *“Do you feel that these substances are available? Ha, ha, ha, yeah, very easy to get; if you want, give me only 2 min to go out and I will buy chewing tobacco and return.”* ***B12:*** *“Drugs are available and easy to obtain, and they exist everywhere. You should only have money.”* ***B2:*** *“Alcohol is also used. They buy it from big shops, even though they are under the age of 18, but they are looking for someone over age to buy it for them.”* ***F14:*** *“First, these substances are available, especially near to schools, such as cigarettes, snuff tobacco, edible cannabis, le,* etc.*”* ***F13:*** *“These substances are available everywhere. Cannabis and shisha cafes are spreading everywhere.”* ***M.T7:*** *“Because the environment around the school has become promising for these behaviors. Also, drug dealers are present in abundance, and all kind of drugs can be found easily.”* ***M.T5:*** *“There is also the availability of drugs and alcohol, which are easy to get by adolescents.”*
	Ex. 21	***B10:*** *“There are unsupervised places near the school where teenagers gather to use all kinds of drugs.”* ***B12:*** *“You can find drug dealers sitting in cafes near the school. There are also places around the school that are not kept, for example, a cellar where students gather to use drugs.”* ***F15:*** *“Lack of control by the authorities concerned with combating drugs and alcohol.”* ***F3:*** *“Alcohol and drugs are sold to under-age teenagers because the law does not apply.”* ***M.T3:*** *“There is a deficiency in security, not only the role of parents; we see that these phenomena happen in full view of the authorities; everything happens and they know about it and they have to punish these dealers.”*
Social Norms	Ex. 22	***B2:*** *“Some parents know that their children smoke, but they don’t do anything to them and consider that as normal.”* ***G4:*** *“In addition, the environment where teens live influences them because they see that most people take drugs. So, they will try to use these substances because it has become usual.”* ***M2:*** *“The society in which teens live is normalized with the use of drugs, and of course, in this case, they will be negatively affected.”* ***F15:*** *“Normalization with these behaviors has an adverse effect on adolescent substance use.”* ***M.T1:*** *“If these behaviors have become so frequent and habitual, the adolescent says he is not alone (a sort of normalization with these behaviors), so this is one of the main reasons for substance use by adolescents.”*
	Ex. 23	***G14:*** *“I think religious beliefs and practices protect against these behaviors; God Almighty has forbidden us all that destroy the mind.”* ***G11:*** *“The absence of religious beliefs in adolescents is a major reason for their tendency toward drugs because the religious education has a crucial role in providing them with immunity from these dangerous behaviors.”* ***F14:*** *“I know a teenager who lived without parents in bad circumstances; despite all this he did not deviate because his religious education was good. I consider that religious and moral education can deal with these problems experienced by adolescents in this period.”* ***F16:*** *“The absence of religious beliefs is a reason for drug abuse.”* ***M.T7:*** *“The causes of these behaviors are intertwined and varied, including the weakness of religious beliefs and the absence of faith and educational immunity of the student.”*

*G girl, B boy, F father, M mother, M.T male teacher, F.T female teacher*

### Perceived benefits of substance use (individual level)

This individual-level theme explains personal factors that cause substance use in adolescents because of perceived beneficial effects. In this theme, we found two sub-themes (personal positive appeal and escape negative state).

***Personal positive appeal*** reflects personal motivations for substance use. Participants in all three FGD categories perceived that a self-image of maturity and looking dangerous were motives for substance use by teens (Ex. 1, Table 3). Others claimed that gaining attention and appreciation from friends were motivating factors (Ex. 2, Table 3), and several stated that having fun and getting high were motivators for substance use (Ex. 3, Table 3). Participants agreed that vulnerability and curiosity during adolescence, a time of increased physical, emotional, social, and behavioral changes, had major roles in substance use initiation among adolescents (Ex. 4 and Ex. 5, Table 3).

Some adolescents leaned on substances to enhance concentration in school. A father stated, “Some people think that using cigarettes help them to focus on their studies, which means they smoke to do well at mathematics for example.” Other adolescents earned money by selling drugs to their peers, as observed by one teacher: “Some adolescents gain money from selling drugs. At the beginning, they offer these drugs to their peers at no cost, until they become addicted.”

***Escape negative states*** reflects the use of substances as a way to manage and forget psychological, personal, and family problems. Use of substances to self-treat depression, stress, anxiety, and lack of self-esteem was reported by several participants, including parents and teachers (Ex. 6 and Ex. 7, Table 3). Many participants viewed that adolescents used substances to forget and bring relief from problems like poverty, divorce, domestic violence, and social conflict. Some participants stated that academic failure was a trigger for teenagers to use substances (Ex. 8, Table 3).

### Awareness and beliefs (individual level)

This theme reflects the protective factor of awareness about the harmful effects of substance use, which can lead to improved resilience and promote positive beliefs against their use. Adolescents and parents stated that awareness gained through personal interactions with family members, schools, and media served as a protective factor against substance use (Ex. 9, Table 3). Teachers agreed that the school setting is important for raising awareness and resistance to drugs but is still insufficient because of the lack of interest for this issue in the school curriculum and the lack of security in the school environment (Ex. 9, Table 3).

The negative influences of media and the lack of awareness about the severity of substance use-related harms, whether in family or in school, were regarded by many participants as important risk factors that increase adolescent substance use (Ex. 10, Table 3). In addition, participants noted the lack of specialists in schools to increase awareness and provide counseling for adolescents. One teacher expressed, “Some addicted adolescents cannot give up. There is also a lack of specialized human resources, such as a psychiatrist or social assistant, at school to follow a smoker or a drug user.”

Some adolescents, through vivid examples of drug-related harms or their desire to maintain their health, shared strong internal beliefs that these substances are not useful as they do not solve problems but destroy health. Parents and teachers also supported that a strong conviction against substance use and a desire to maintain health are protective factor (Ex. 11, Table 3). For example, adolescents who can organize their free time by filling it with beneficial activities such as sports are protected from being involved in substance use (Ex. 12, Table 3).

### Family influence (relationship level)

This theme reflects influences of family, which may be either a protective or a risk factor for substance use in adolescents. In all three FGDs, most participants emphasized that adolescents who used substances had poor parenting, especially when their parents or siblings were substance users, thus implicitly influencing decisions to use drugs through observation or modeling. Adolescents agreed that, if parents used substances, their children will be much more likely to be involved in the same behavior (Ex. 13, Table 3).

Lack of parental supervision, lack of affection, and neglect within the family were expressed by some participants as risk factors for substance use (Ex. 14, Table 3). Participants believed that parents must be more supportive and embracing and less neglectful of their children’s behaviors.

In contrast, other adolescents stated that restriction and control from parents can lead to a lack of self-esteem, prompting use of drugs as self-protective or an act of rebellion against this control. Because teenagers often crave independence, they believed that strict and severe parental rules could actually backfire against the prevention of substance use. As stated by a male adolescent, “In some cases, you find that parents over-control teenagers, so they do what they want, because more control gives the opposite result. Parental control must be carried out intelligently without abuse. For example, if my father treated me with extra-rigor, I wouldn’t do what he asked me, but if he joked with me and treated me kindly, I would listen.”

In the three FGDs, most participants agreed that excess or limited pocket money can influence risky behaviors, including substance use (Ex. 15, Table 3). In contrast, having shared culture and beliefs in families against controlling behaviors are effective in preventing substance use in adolescents, particularly if there is a high level of parental monitoring. However, adolescents agreed that parental control must be accompanied by mutual trust, which is gained through meaningful discussions to understand unhealthy behaviors of their children. Intimate discussions may cause teenagers to avoid disappointing their family members and losing their confidence. This idea was supported by many adolescents, parents, and teachers (Ex. 16, Table 3).

Having a father who smokes could lead to advice regarding the negative health effects of smoking. One male adolescent noted, “My father smokes; he is the one who told me not to accompany friends who smoke because he was a victim when he was young and wants me not to be like him.”

### Peer influence (relationship level)

Substance use in friends was considered by adolescents to be an important risk factor for experimenting and using substances, a view supported by parents and teachers (Ex. 17, Table 3). The desire to fit in and be accepted into a particular peer group can lead to drug- taking behavior. One adolescent stated, “When you find yourself in a group of smoking friends. When I tried to smoke for the first time it was only in front of my friends, I wanted to tell them that I share the same activities with you.” This dominant factor of peer influence was mentioned in narratives of all participants.

Some adolescents said that lack of money was not a deterrent for substance use because they can obtain drugs from friends or make some drugs themselves (e.g., edible cannabis products). An adolescent stated, “Some drugs, like edible cannabis, students make it here in school, so they agree that each one comes with a certain ingredient and then make it themselves.” Adolescents and teachers also mentioned the role of emotional relationships between boys and girls, which can influence the use of drugs by adolescents, especially girls under the influence of their boyfriends (Ex. 18, Table 3).

In contrast, in all FGDs, most participants believed that having close friends who do not have such behaviors can protect adolescents from this risk (Ex. 19, Table 3).

### Easy accessibility of substances (community level)

This theme was based on statements in which substances, despite being illegal, were widely available and easily accessible for adolescents. Drugs could be obtained free of charge from friends or with their pocket money. Places selling substances and places for users were stated to be abundant and found everywhere (Ex 20, Table 3).

Many adolescents, parents, and teachers expressed that there is a lack of laws that penalize drug dealers and users. In addition, schools do not provide adequate surveillance, and there are unguarded places near the school where adolescents meet to take substances (Ex. 21, Table 3).

### Social norms (societal level)

This theme was based on the negative or positive influence of collective social norms, such as normalization and religious beliefs, on substance use in adolescents.

In terms of normalization**,** participants stated that substance use appears to be integrated into the daily lives of people and has become more accepted by society. This, coupled with the desire to be like others, could put adolescents at a high risk of engaging in this risky behavior (Ex. 22, Table 3). Many participants viewed strong religious beliefs as a crucial protective factor that can help adolescents to avoid substances (Ex. 23, Table 3).

## Discussion

This study aimed to better understand factors influencing substance use behaviors in Moroccan adolescents using a qualitative approach and the socio-ecological model as a theoretical framework. In accordance with previous ecological theories of development [28, 29], our overarching themes highlight several contextual risk and protective factors that influence substance use in adolescents. These themes were within all four socio-ecological levels **(**individual, relationship, community, and societal).

At the individual level, the theme of perceived benefits of substance use (reflecting beliefs on the beneficial effects of substances, including cannabis, cigarettes, and edible cannabis products) was consistent with previous qualitative investigations [14, 30]. This theme comprised two sub-themes: personal positive appeals and escape negative states. The personal positive appeal sub-theme includes boosting self-image, having fun, experimentation, and other normal adolescent behaviors [2, 30]. Perceived benefits can also include financial ones. In agreement with a previous study, our study showed that many adolescents were motivated to get pocket money by dealing drugs [31]. The use of substances in adolescents due to perceived benefits can be explained by the core construct in Bandura’s social cognitive theory, which suggests that behavior is partly motivated by the anticipated consequences, or outcome expectations, of the behavior [32]. Thus, the greater the perceived benefit associated with a behavior, the more likely that the behavior will be adopted.

The second sub-theme, escape of negative states, reflects the use of drugs to flee undesirable situations, especially those caused by psychological and family problems [14, 30, 33]. Substance use can be coupled with psychological problems, including suicidal ideation in Moroccan adolescents [34]. Evidence points to significant associations between psychological problems and substance use in adolescents [1, 7, 10, 23]. Adolescents may use drugs to escape or find relief from troubles and stressful situations or as a way to manage their problems. This self-medication theory of the use of psychoactive substances to self-regulate or alleviate stress [35, 36] offers an understanding of this finding. Therefore, a psychoeducational prevention program should provide adolescents with effective ways to deal with stress and life problems during adolescence, which could perhaps decrease substance use in adolescents.

The theme of awareness and beliefs was both protective against and a risk for substance use. Similar to previous studies, we found that lack of awareness about substance use-related harms was an important risk factor for adolescent substance use [19, 37]. Lack of knowledge regarding substance use may be due to poor dissemination within families or within schools. This is heightened by influences from media, which often present favorable images of tobacco and alcohol through advertising or “normalize” its presentation [37, 38]. In contrast, strong positive beliefs against substances can be a protective factor [19]. Messages from schools and media, personal interactions with family members, and vivid examples showing the harmful effects of drugs can influence personal beliefs [19, 37]. Nevertheless, our surveyed teachers mentioned that messages and education from schools were not sufficiently addressing drug awareness or promoting positive beliefs; teachers mentioned lack of health education in the school’s curriculum and absence of extracurricular activities. Drug prevention in adolescents can greatly benefit from the incorporation of substance use education into school curriculum, specifically including the use of modern technology to improve student participation [33, 39]. In addition, having extracurricular activities in schools and encouraging the intense participation of adolescents in these activities have been shown to reduce substance use in adolescents [40].

From these observations, better-designed intervention program should both increase adolescents’ knowledge about substance use-related hazards and change adolescents’ misconceptions about the perceived benefits of substance use. In addition, programs should include ways to develop adolescent resiliency with regard to decision-making, positive thinking, and coping abilities.

At the relationship level, we found that family and peers play important influential roles in substance use in adolescents, which is consistent with previous social and environmental theories [29, 41]. Similar to other studies [19, 37, 42, 43], our participants identified poor family relationships, lack of control or over-control, lack of affection, unmonitored pocket money, and substance use behaviors of parents and siblings as potential risk factors in adolescents. In a study of parenting styles in several European countries, neglectful parenting (neither warmth nor strictness) and authoritarian parenting (strictness but not warmth) were associated with high levels of substance use in adolescents. However, when parenting styles included an environment of acceptance, dialogue, and affection, levels of substance use were lower [44]. Substance-using family members who are role models, especially parents and siblings, can negatively influence the use of substances in adolescents [15, 37, 43]. Children who grow up with parents or siblings who use drugs may model behaviors and attitudes regarding substance use; through social learning processes of parental acceptance, the likelihood of substance use initiation and continuation is increased [45]. In contrast, and similar to other studies [19, 30, 37, 42, 43], we found that family beliefs against drug use are effective in preventing substance use in adolescents. Such results support the strong association between parental bonding and substance use in Moroccan adolescents from quantitative findings (unpublished data). Establishing a healthy culture and environment for adolescents can help protect against substance use. Therefore, intervention strategies should also include information regarding positive parenting practices and should educate substance-using family members about the negative effects of substance use on adolescents and the family in general [43].

Participants in our study emphasized that peers can either increase or decrease substance use among adolescents. As already documented, student drug users generally seek to attract other people to use substances in schools; under this pressure, adolescents may use substances to fit in with peers [14, 19, 30, 46]. Furthermore, as reported in a previous quantitative study (unpublished data), there is a strong association between substance use in adolescents and substance use behaviors of their friends. This influence is particular important during the adolescent developmental stage, where teens begin the process of differentiating themselves from their parents and orienting toward their peers; during this period, they also tend to prioritize the values, attitudes, and behaviors of peers versus those of their own family [47]. The role of peers in inducing substance use by adolescents appears to be moderated by the poor role of parents [30, 46]. In our study, participants stated that peers were often the primary way to obtain drugs or make certain drugs with local ingredients (edible cannabis products), as reported elsewhere [19]. In addition, our participants suggested that emotional relationships between girls and boys can play a role in substance use initiation, especially for girls who receive pressure from boyfriends or as a coping mechanism during relationship disturbances [48]. In contrast, participants also believed that having close friends who make good choices and those not engaged in high-risk activities could act as a protective factor against substance use, as documented previously [19, 30, 39]. Peers who do not engage in drug use behaviors and have negative attitudes toward the use of substances are likely to influence similar behaviors and attitudes. Consequently, it is important to provide adolescents with ways to counteract negative peer influences, for example, by providing effective refusal skills to withstand peer pressure and using prosocial peers in the implementation of substance use prevention programs.

Accessibility and affordability of substances have been noted in several studies as risk factors for substance use in adolescents [14, 19, 30, 37]. Our participants reported that substances were widely available and easily accessible in their environment despite their illegality. In addition, most teens knew where to easily obtain substances at lower prices. It is rational to suggest that, when more drugs are available in a given society, there is more probability that adolescents engage in drug use and other risky behaviors. Thus, minimizing the availability of substances by strengthening the security in school environments and imposing tougher laws to restrict substance delivery, especially among adolescents, may contribute to decreasing risky behaviors of adolescents.

At the societal level, we found that normalization of substance use, that is, integration of drugs into daily lives and drugs becoming tolerated by society, negatively affected risky behavior in adolescents. This finding was in line with other studies [30, 49]. In a study on how social norms influence adolescent substance use, descriptive norms (perceptions of what others do) were significantly associated with substance use in adolescents [50]. Thus, the widespread use of substances in young people (i.e., descriptive norms) may send a subtle message that this behavior is accepted and already expected and encourage other adolescents to engage socially in substance use [50]. On the other hand, our participants regarded strong religious beliefs as a protective factor against substance use in adolescents, although some mentioned that poor religious beliefs could also be a risk factor for substance use. This protective relationship between religiosity and health behavior, especially substance use, among adolescents has been well established in the literature [19, 42, 51]. The implementation of social norm interventions that attempt to modify perceptions of what behavior is normative could be a way to influence actual behavior. In addition, health interventions for adolescents should consider including religion outlets as a way to raise awareness of the risks associated with substance use.

### Strengths and limitations

This study was conducted in two middle schools in an urban area of Morocco. However, our aim was not to generalize the findings; rather, our objective was to obtain a greater understanding of contextual risks and protective factors of substance use in adolescents. In addition, this first qualitative study in Morocco allowed us to better understand the different influencing factors on substance use in these adolescents. The large number of participants, the triangulation of participants, the theoretical framework, and the rigorous methodology applied allowed a more comprehensive overview.

## Conclusions

Participants in this study recognized a number of perceived risks of and protective factors against substance use in adolescents. These results emphasize that effective prevention programs need to address multiple levels, from the individual to the societal, and should address the social norms and the government policies toward adolescent substance use. Furthermore, inclusion of intrapersonal factors, such as raising awareness about the harmful effects of drugs and building coping abilities and refusal skills in adolescents against risky behaviors, can result in a more favorable school-based prevention strategy. Finally, introducing health education as a school subject in the Moroccan Education Curriculum is a need for adolescents, which can serve as a way to enhance healthy behaviors in adolescents.

## Abbreviation

FGDs: Focus group discussions
